# Supplementary material for: The interactome of intact mitochondria by cross-linking mass spectrometry provides evidence for coexisting respiratory supercomplexes
Source: Mol Cell Proteomics. 2017 Dec 8;17(2):216–32. doi: 10.1074/mcp.RA117.000470 (PMC5795388; doi:10.1074/mcp.RA117.000470)
Supplement: Supplemental Data [file supp_RA117.000470_133922_0_supp_23978_fzffwf.zip › spectra_annotation/mito_DR_spectra_annotation/417-1-1-1-3-1.pdf]

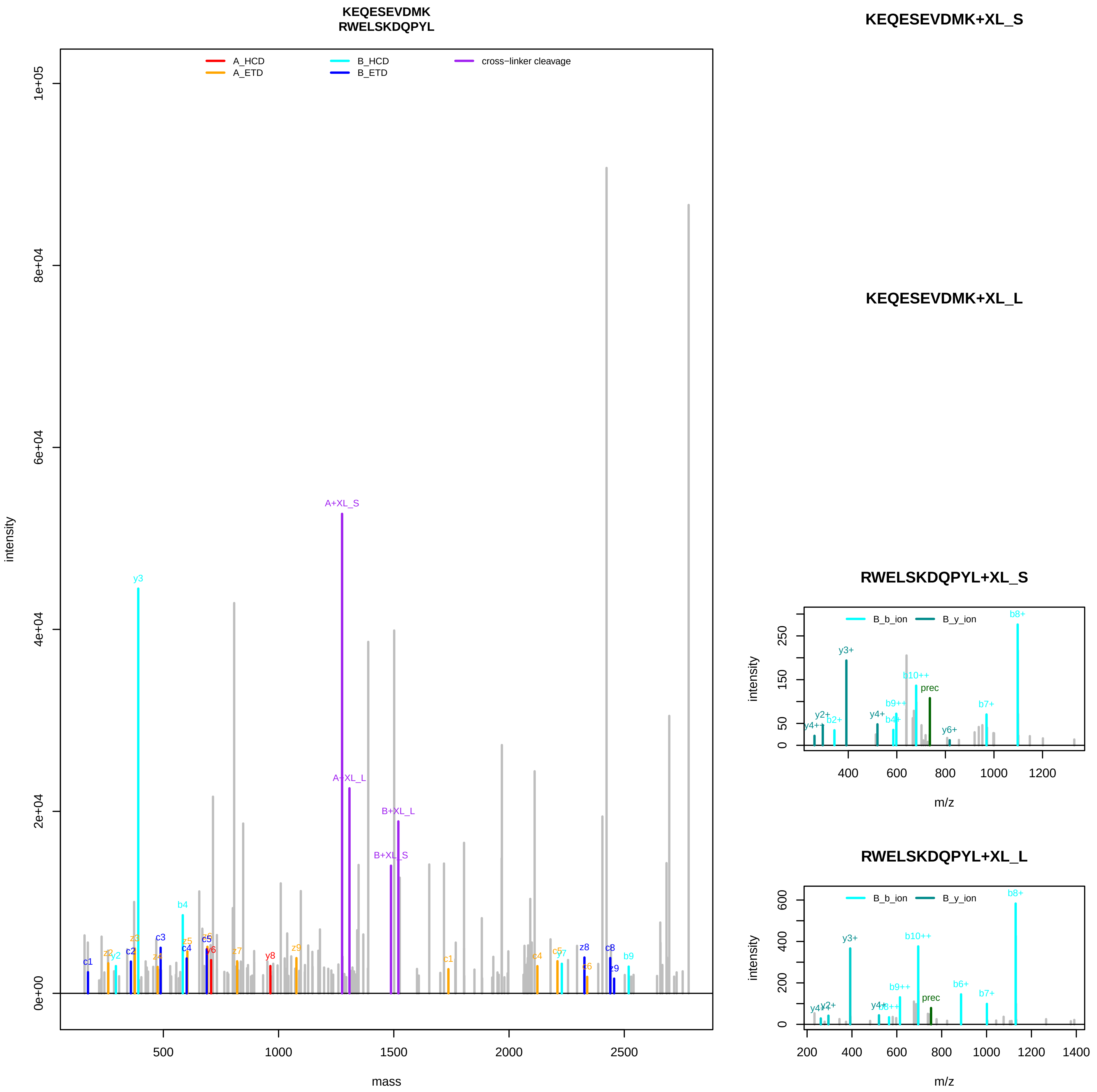
KEQESEVDMK
RWELSKDQPYL
A_HCD
A_ETD
B_HCD
B_ETD
cross−linker cleavage
intensity
mass
0e+00
2e+04
4e+04
6e+04
8e+04
1e+05
500
1000
1500
2000
2500
A+XL_S
A+XL_L
B+XL_L
B+XL_S
y3
b4
c1
c2
c3
c4
c5
c8
y2
y6
y7
y8
z2
z3
z4
z5
z6
z7
z8
z9
c1
c4
c5
c6
b9
KEQESEVDMK+XL_S
KEQESEVDMK+XL_L
RWELSKDQPYL+XL_S
B_b_ion
B_y_ion
intensity
m/z
0
50
150
250
400
600
800
1000
1200
b8+
y3+
b10++
prec
b9++
y2+
b2+
y4+
b4+
b7+
y6+
y4++
RWELSKDQPYL+XL_L
B_b_ion
B_y_ion
intensity
m/z
0
200
400
600
200
400
600
800
1000
1200
1400
b8+
y3+
b10++
b9++
b6+
b7+
prec
y4++
y2+
y4+
b8++
